# Supplementary material for: Effects of additional oral theophylline with inhaled therapy in patients with stable chronic obstructive pulmonary disease: A systematic review and meta-analysis
Source: PLoS One. 2025 May 6;20(5):e0321984. doi: 10.1371/journal.pone.0321984 (PMC12054895; doi:10.1371/journal.pone.0321984)
Supplement: S1 File — (DOCX) [file pone.0321984.s005.docx]

Response to editor the question of how missing data were handled:

Indeed, Some research subjects may withdraw from the study during the research process and we perform the analysis in accordance with the intention-to-treat principle.
