# Supplementary material for: Effects of additional oral theophylline with inhaled therapy in patients with stable chronic obstructive pulmonary disease: A systematic review and meta-analysis
Source: PLoS One. 2025 May 6;20(5):e0321984. doi: 10.1371/journal.pone.0321984 (PMC12054895; doi:10.1371/journal.pone.0321984)
Supplement: S2 File — (DOC) [file pone.0321984.s006.doc]

| **Section/Topic** | **#** | **Checklist Item** | **Reported on Page #** |
| --- | --- | --- | --- |
| **TITLE** | | | |
| Title | 1 | The report was Identified as a systematic review and meta-analysis | 1 |
| **ABSTRACT 2** | | | |
| Structured summary | 2 | The structured abstract includes objective、data sources、method、results、conclusion and limitations. | 1,2 |
| **INTRODUCTION** | | | |
| Rationale | 3 | the rationale for the review was described in introduction. | 2 |
| Objectives | 4 | The statement of questions was described in introduction. | 2 |
| **METHODS** | | | |
| Protocol and registration | 5 | There was no protocol and registration | Non |
| Eligibility criteria | 6 | The study characteristics and report characteristics as eligibility criteria were described. | 3 |
| Information sources | 7 | The information sources in the search were described. | 2 |
| Search | 8 | search strategy was described. | 3 |
| Study selection | 9 | the process for selecting studies was stated. | 3 |
| Data collection process | 10 | Describe method of data extraction from reports. | 3 |
| Data items | 11 | Data items were listed. | 3 |
| Risk of bias in individual studies | 12 | Describe methods used for assessing risk of bias of individual studies. | 3 |
| Summary measures | 13 | State the principal summary measures. | 3 |
| Synthesis of results | 14 | Describe the methods of handling data and combining results of studies. | 3 |

Page 1 of 2

| **Section/Topic** | **#** | **Checklist Item** | **Reported on Page #** |
| --- | --- | --- | --- |
| Risk of bias across studies | 15 | Specify any assessment of risk of bias that may affect the cumulative evidence | 3 |
| Additional analyses | 16 | There were no additional analyses. | Non |
| **RESULTS** | | | |
| Study selection | 17 | Give numbers of studies screened, assessed for eligibility, and included in the review, with reasons for exclusions at each stage, with a flow diagram. | 4 |
| Study characteristics | 18 | For each study, present characteristics for which data were extracted and provide the citations. | 5,6 |
| Risk of bias within studies | 19 | Present data on risk of bias of each study. | 7 |
| Results of individual studies | 20 | For all outcomes were presented with forest plot. | 7,8,9,10 |
| Synthesis of results | 21 | Present the main results of the review. | 7,8,9,10 |
| Risk of bias across studies | 22 | Present results of any assessment of risk of bias across studies. | 7 |
| Additional analysis | 23 | There were no additional analyses. | Non |
| **DISCUSSION** | | | |
| Summary of evidence | 24 | Summarize the main findings and the findings were explained. | 11,12,13 |
| Limitations | 25 | The limitation was presented in discussion. | 13 |
| Conclusions | 26 | The conclusion was provided | 13 |
| **FUNDING** | | | |
| Funding | 27 | There was no funding support. | Non |

From: Page MJ, McKenzie JE, Bossuyt PM, et al. The PRISMA 2020 statement: an updated guideline for reporting systematic reviews. BMJ 2021;372:n71. doi:10.1136/bmj.n71

For more information, visit: www.prisma-statement.org.

Page 2 of 2
