# Supplementary material for: Effects of additional oral theophylline with inhaled therapy in patients with stable chronic obstructive pulmonary disease: A systematic review and meta-analysis
Source: PLoS One. 2025 May 6;20(5):e0321984. doi: 10.1371/journal.pone.0321984 (PMC12054895; doi:10.1371/journal.pone.0321984)
Supplement: Attachment — Submitted filename: Response to Reviewers.docx [file pone.0321984.s008.docx]

Dear Editor and Reviewers:

Thank you for your letter and for the comments concerning our manuscript entitled “Effects of additional oral theophylline with inhaled therapy in patients with stable chronic obstructive pulmonary disease: A systematic review and meta-analysis”. Those comments are all valuable and very helpful for revising and improving our paper, as well as the important guiding significance to our researches. We have studied comments carefully and have made correction which we hope meet with approval. Revised portion are marked in red in the paper. The main corrections in the paper and the responds are as flowing:

Reviewer #1: The manuscript by Yang et al presented a systematic review and meta-analysis on the effects of additional oral theophylline in conjunction with inhaled therapy in stable COPD patients. The topic is an interesting one in clinical relevance as theophylline is applied in COPD for very long time. The study is well-structured, follows the PRISMA guidelines, and uses standard meta-analysis techniques. However, there are areas that require clarification and further major revision before it get accepted for publication.

(1) The abstract should mention the increased adverse reactions observed to avoid overstating the potential benefits of theophylline.

Respond: thank you for reviewer's comments. We have added the mention in abstract.

(2) The manuscript addresses an important question regarding the role of theophylline as adjunct therapy in COPD, but the introduction should provide more justification for this research, considering known risks of theophylline.

Respond: thank you for reviewer's comments. Indeed, emphasizing the risks of theophylline is very important and could further illustrate the importance of our research. So, we have added relevant content in the introduction.

(3) Significant heterogeneity was observed in key outcomes (e.g., symptom scores, adverse reactions). The manuscript should address potential sources and perform subgroup analyses to explore this further.

Respond: thank you for reviewer's comments. I am so sorry for my mistake that I mistakenly labeled the meta-analysis figure of adverse reactions. I have corrected it in manuscript and there was no Significant heterogeneity in adverse reactions. Indeed, there was Significant heterogeneity in symptom scores. The symptom scores were subjectively evaluated and recorded by researchers and there were differences between different studies that may lead to significant heterogeneity. It is difficult to perform subgroup analyses because of that It is difficult to classify the included literature.

(4) The manuscript needs to better discuss the clinical risks posed by the high rate of adverse reactions, particularly in the context of theophylline’s known narrow therapeutic window.

Respond: thank you for reviewer's comments. We have added the discussion in manuscript about the clinical risks posed by the high rate of adverse reactions as following: The therapeutic window of theophylline is relatively narrow (5~20 μg/ml) and show high rate of adverse reactions even though within the therapeutic window (15~20 μg/ml) such as nausea、vomiting、irritability and insomnia. When the serum concentration exceeds 20μg/ml, tachycardia and arrhythmia may occur. Fever、dehydration、convulsions and even death caused by respiratory and cardiac arrest may occur when serum concentration exceeds 40μg/ml. In addition, many factors could affect the serum concentration of theophylline, such as age、medication and disease. Therefore, clinician need to be in cautions when prescribing theophylline and it is recommended to monitor the serum concentration.

(5) The non-significant results for FEV_1_/FVC% and symptom scores should be more clearly interpreted to guide clinicians on their clinical relevance.

Respond: Thank you for reviewer's comments. Our meta-analysis indicated non-significant results for FEV_1_% pred、FEV_1_/FVC% and symptom scores. FEV_1_% pred and FEV_1_/FVC% were important index to evaluate lung function and the effectiveness of medication therapy. Theophylline is a weak acting bronchodilator and adding theophylline on the basis of potent inhaled bronchodilators may do not produce additional effects, inducing no improvement in FEV_1_% pred and FEV_1_/FVC%. Symptoms scores are closely related to lung function and non-significant FEV_1_% pred and FEV_1_/FVC% improvements result non-significant symptom scores. The above content was added into the discussion of the manuscript.

(6) More details on how comorbidities were handled in the included studies are necessary for transparency in the methodology.

Respond: Thank you for reviewer's comments. Indeed, comorbidities may affect the effectiveness of COPD treatment. The included studies excluded patients with comorbidities that could affect the evaluation of treatment efficacy, such as bronchiectasis、tuberculosis、cancer、heart failure and infections. For some comorbidities, such as hypertension、osteoporosis, it is expected that they will not obviously affect the evaluation of treatment efficacy and have not been excluded, and there is no baseline characteristic difference between the treatment group and the control group.

（7）The lack of allocation concealment in some studies should be thoroughly discussed as a limitation that could affect the robustness of the findings.

Respond: Thank you for reviewer's comments. Allocation concealment is an important step in randomized controlled trials, aimed at ensuring the confidentiality of the randomization process and preventing researchers from predicting the grouping of subjects before allocation, thereby reducing selection bias. If no allocation concealment scheme, researchers may make subjective judgments during randomization, leading to certain subjects being preferentially assigned to specific groups and resulting in selection bias. The results of meta-analysis depend on the quality of the included studies and the reliability of the randomization process. The lack of allocation concealment schemes in the original research would affect the objectivity and accuracy of the meta-analysis. The above content was added into the discussion of the manuscript.

(8) The discussion should be expanded to clarify where, if at all, theophylline should fit in current COPD treatment guidelines, especially given the availability of more effective therapies.

Respond: Thank you for reviewer's comments. Our Meta analysis results show that adding theophylline to inhalation therapy can reduce the risk of acute exacerbation in patients with COPD. Therefore, the theophylline may be more suitable for patients who have a high risk of acute exacerbation especially when have already been treated with ICS+LABA+LAMA.

(9)The conclusion should highlight specific research gaps, such as the need for studies on long-term outcomes or focusing on different COPD phenotypes.

Respond: Thank you for reviewer's comments. We have added relevant contents in the conclusion of the manuscript.

Reviewer #2:

1. What are the inclusion and exclusion criteria for patients with COPD with/without other complications? Needs to be well defined.

Respond: Thank you for reviewer's comments. The inclusion criteria included:(1) stable COPD; (2) age>18 years old; (3) RCTs; (4) no limitations in gender, age, ethnicity, or treatment duration. (5) without bronchiectasis、tuberculosis、cancer、heart failure and infections. Exclusion criteria included: (1) non-stable COPD; (2) non-RCTs；(3) Valid ending data unable to be extracted; (4) Full text of the study is not available. (5) with bronchiectasis、tuberculosis、cancer、heart failure and infections. We have added in the manuscript.

2. Table 2 needs to be re-looked into. There are mismatches between cardiovascular and neurological adverse reactions. Besides these common symptoms, adverse reactions, and drug-drug interactions may be included.

Respond: Thank you for reviewer's comments. I am so sorry for my mistake of mismatching between cardiovascular and neurological adverse reactions. We have corrected it in the manuscript. Theophylline is metabolized by cytochrome P450 mixed function oxidases. Many other drugs can modify theophylline. For example, macrolides and quinolones could decline the clearance of theophylline and may lead to an increase of serum concentration, rising the risk of adverse reactions of theophylline. The content above was added in the discussion of the manuscript.

3. Many times, COPD patients are treated with other drugs, including antibiotics. Such complications can be discussed in the discussion.

Respond: Thank you for reviewer's comments. Stable COPD generally does not require antibiotics. When COPD patients experience acute exacerbation, short-term use of antibiotics is possible and is not expected to have a significant impact on research.
