# Supplementary material for: Effects of additional oral theophylline with inhaled therapy in patients with stable chronic obstructive pulmonary disease: A systematic review and meta-analysis
Source: PLoS One. 2025 May 6;20(5):e0321984. doi: 10.1371/journal.pone.0321984 (PMC12054895; doi:10.1371/journal.pone.0321984)
Supplement: Attachment — Submitted filename: Response to Reviewers_.docx [file pone.0321984.s009.docx]

Dear Editor and Reviewers:

Thank you for your letter and for the comments concerning our manuscript entitled “Effects of additional oral theophylline with inhaled therapy in patients with stable chronic obstructive pulmonary disease: A systematic review and meta-analysis”. Those comments are all valuable and very helpful for revising and improving our paper, as well as the important guiding significance to our researches. We have studied comments carefully and have made correction which we hope meet with approval. Revised portion are marked in red in the paper. The main corrections in the paper and the responds are as flowing:

Reviewer#3: The authors have addressed most of the comments and significant improvement in the manuscript. However, I request the authors further clarify and refine before its get accepted for publication.

(1) The author has acknowledged heterogeneity in symptom scores, the justification for not performing subgroup analyses could be expanded. If subgroup analyses are truly infeasible, please consider alternative approaches, such as sensitivity analyses or meta-regression, to explore the sources of heterogeneity.

Respond: thank you for reviewer's comments. When conducting a meta-analysis of symptom scores, only three studies were included, making subgroup analysis difficult. Sensitivity analysis using Stata14 software revealed that after excluding the Subramanian2015 study, the heterogeneity was significantly reduced, yet there remained no significant difference in symptom scores between the two groups (MD = -0.14, 95% CI: -0.44 to 0.15, p = 0.35, I² = 37%)（As shown in the figure below). The study of Subramanian2015 specifically reported night symptom scores, which may explain the heterogeneity compared to the other two studies. We have added the content in the discussion section in manuscript.

(2) It has been mentioned difficulties in classifying literature for subgroup analysis. It would be helpful to elaborate on these difficulties in the methods or discussion section. For example, were there inconsistencies in study design, population characteristics, or outcome measures that prevented meaningful subgroup comparisons.

Respond: thank you for reviewer's comments. Indeed, meaningful subgroup analyses were not conducted due to challenges in classifying the literature with regard to inconsistencies in study design, population characteristics, and medication regimens.We have added the content in the discussion section in manuscript.

(3) The authors have improved the discussion regarding the non-significant changes in lung function parameters (FEV_1_/FVC%), further elaboration on the clinical implications would be beneficial. How should clinicians interpret these findings in the context of COPD management, particularly in patients who are already receiving triple therapy?

Respond: thank you for reviewer's comments. FEV_1_/FVC%<70% serves as a key diagnostic criterion for COPD. A persistent decline in FEV_1_/FVC% during follow-up monitoring of COPD also indicates progressive deterioration of lung function. Meta-analysis findings demonstrate that the addition of theophylline to inhaled therapy do not significantly improve FEV_1_/FVC%. Although theophylline exhibits bronchodilatory effects, its efficacy is relatively modest. When combined with novel potent bronchodilators (e.g., LABA and LAMA) in COPD treatment regimens, theophylline may not exhibit additional bronchodilation and therefore fails to improve pulmonary function parameters. However, meta-analyses have demonstrated that adjunctive theophylline therapy may reduce acute exacerbation risks, primarily attributable to its anti-inflammatory properties rather than bronchodilatory mechanisms. Therefore, theophylline is recommended for patients with high risk of acute exacerbations, particularly for those already on triple therapy, but is not recommended for pulmonary function enhancement in COPD management.

(4) The authors have acknowledged the lack of allocation concealment in some studies as a potential limitation, which is appreciated. However, a brief discussion on how this might have influenced your results (e.g., overestimation of theophylline’s benefits) would further enhance transparency.

Respond: thank you for reviewer's comments. The absence of allocation concealment in primary studies may systematically compromise the validity of meta-analysis, particularly through inflated treatment effect estimates (e.g., overestimation of theophylline’s benefits) . We have added relative content in the manuscript according to reviewer's suggestion.

(5) The conclusion has added the research gaps. However, adding more specific recommendations (e.g., the need for long-term studies assessing cardiovascular risks or theophylline's role in different COPD phenotypes) would make the conclusion and manuscript for further improvement.

Respond: thank you for reviewer's comments. We have added relative content in the manuscript according to reviewer's suggestion.
